# Gleanings

**Published:** 1898-01

**Authors:** 


### GLEANINGS.

Dr. J. J. Drasky, of Crete, Nebraska, reports successful results in treating "Stricture of the Teat" with electricity.

One of the Boston Fire Department's horses, after fifteen years of continuous service, has been pensioned off, and will enjoy the balance of his days in peace and amid plenty.

A recent number of *The Breeder and Sportsman* has the following paragraph :

Dr. Isaac W. O'Rourke, city veterinarian of San Francisco, has discovered a new disease among horses. It appeared only recently, but is spreading over the State with remarkable rapidity, particularly on the lands adjoining the San Joaquin and Sacramento Rivers. Dr. O'Rourke went to Tubbs' Island, on the San Joaquin River, last Wednesday, to inspect two hundred horses belonging to William Tubbs, the San Francisco cordage manufacturer. He found fifty of the animals suffering from the mysterious disease. It manifests itself in the form of abscessed swellings on different parts of the body. Some of these attain an enormous size. So far the number of deaths resulting from the malady has been small. It is Dr. O'Rourke's opinion that the disease grows out of fly-bites, and was introduced there by foreign stock of the poisonous fly, which is a stranger in California, is smaller than the common house-fly, and yellow in color. For the treatment of the disease the veterinarian recommends compound camphor liniment.

One of the most curious trades extant is that of a man in Berlin who gets a living by breeding rats for vivisection purposes.

Horses cannot be hired at any price in Whitman County, Wash., where 1,500,000 bushels of grain were harvested in one week recently. The grain was valued at $1,050,000.

